# Machine-learning-based models for the optimization of post-cervical spinal laminoplasty outpatient follow-up schedules

**DOI:** 10.1186/s12911-024-02693-y

**Published:** 2024-09-30

**Authors:** Yechan Seo, Seoi Jeong, Siyoung Lee, Tae-Shin Kim, Jun-Hoe Kim, Chun Kee Chung, Chang-Hyun Lee, John M. Rhee, Hyoun-Joong Kong, Chi Heon Kim

**Affiliations:** 1 Department of Medicine, Seoul National University College of Medicine, 103 Daehak-ro, Jongno-gu, Seoul, 03080 Republic of Korea; 2 Department of Transdisciplinary Medicine, Seoul National University Hospital, 101 Daehak-Ro, Jongno-gu, Seoul, 03080 Republic of Korea; 3 Innovative Medical Technology Research, Seoul National University Hospital, 101 Daehak-Ro, Jongno-gu, Seoul, 03080 Republic of Korea; 4 School of Medicine, the Faculty of Medical Science, Newcastle University, Newcastle Upon Tyne, NE2 4HH UK; 5 Department of Neurosurgery, Champodonamu Hospital, 32 Baumoe-ro 35-gil, Seocho-gu, Seoul, 03080 Republic of Korea; 6 Department of Neurosurgery, Seoul National University Hospital, 101, Daehak-ro, Jongno-gu, Seoul, 03080 Republic of Korea; 7 Department of Neurosurgery, Seoul National University College of Medicine, 103 Daehak-ro, Jongno-gu, Seoul, 03080 Republic of Korea; 8 Department of Brain and Cognitive Sciences, Seoul National University, 101, 1, Gwanak-ro, Gwanak-gu, Seoul, Republic of Korea; 9 Department of Orthopaedic Surgery, Emory University School of Medicine, Atlanta, GA 30322 USA; 10 Department of Medical Device Development, Seoul National University College of Medicine, 103 Daehak-ro, Jongno-gu, Seoul, 03080 Republic of Korea

**Keywords:** Cervical vertebra, Laminoplasty, Machine learning, Outpatient clinic, Patient outcome assessment, Telemedicine

## Abstract

**Background:**

Patients undergo regular clinical follow-up after laminoplasty for cervical myelopathy. However, those whose symptoms significantly improve and remain stable do not need to conform to a regular follow-up schedule. Based on the 1-year postoperative outcomes, we aimed to use a machine-learning (ML) algorithm to predict 2-year postoperative outcomes.

**Methods:**

We enrolled 80 patients who underwent cervical laminoplasty for cervical myelopathy. The patients’ Japanese Orthopedic Association (JOA) scores (range: 0–17) were analyzed at the 1-, 3-, 6-, and 12-month postoperative timepoints to evaluate their ability to predict the 2-year postoperative outcomes. The patient acceptable symptom state (PASS) was defined as a JOA score ≥ 14.25 at 24 months postoperatively and, based on clinical outcomes recorded up to the 1-year postoperative timepoint, eight ML algorithms were developed to predict PASS status at the 24-month postoperative timepoint. The performance of each of these algorithms was evaluated, and its generalizability was assessed using a prospective internal test set.

**Results:**

The long short-term memory (LSTM)-based algorithm demonstrated the best performance (area under the receiver operating characteristic curve, 0.90 ± 0.13).

**Conclusions:**

The LSTM-based algorithm accurately predicted which group was likely to achieve PASS at the 24-month postoperative timepoint. Although this study included a small number of patients with limited available clinical data, the concept of using past outcomes to predict further outcomes presented herein may provide insights for optimizing clinical schedules and efficient medical resource utilization.

**Trial registration:**

This study was registered as a clinical trial (Clinical Trial No. NCT02487901), and the study protocol was approved by the Seoul National University Hospital Institutional Review Board (IRB No. 1505-037-670).

**Supplementary Information:**

The online version contains supplementary material available at 10.1186/s12911-024-02693-y.

## Introduction

At predetermined intervals following spinal surgery, patients typically visit outpatient clinics for postoperative follow-up. Despite some variations, depending on the institution, doctor, and patient-specific condition, the postoperative follow-up typically includes visits at 1, 3, 6, and 12 months, with annual visits thereafter [1–9]. However, based on their perception that a routine visit is unnecessary, patients sometimes skip scheduled appointments. Moreover, the schedule is not usually individualized by clinical outcomes and is relatively inflexible. For example, despite symptomatic improvement, a patient may continue to adhere to a predetermined hospital visit schedule [1–12]. Systematic modulation of the outpatient visits to individualized clinical outcomes could improve the efficient use of medical resources, and thereby benefit both patients and healthcare providers through optimization of medical resource utilization. Previous algorithms for improving the efficiency of outpatient clinics prioritized patient status [13–19]. Research on clinical history-based prediction of future patient conditions is ongoing [20–23]. Predictive algorithms differ, based on the analytical method, such as machine learning (ML), which have been developed to predict postoperative states [24–30]. As these algorithms may not be applicable to every disease, the creation of customized algorithms for each condition is essential. For example, to predict the treatment outcomes of patients with lumbar disc herniation, Pedersen et al. recently used ML and deep learning by applying decision tree (DT), support vector machine (SVM), random forest (RF), and boosted tree algorithms [29].

Cervical laminoplasty, commonly used to treat cervical myelopathy [2, 4–9, 31–33], involves changes to the configuration of the spinal lamina to expand the cervical spinal canal, which is subsequently maintained using a metal plate/screw system and solid bony fusion at the lamina for approximately 1 year. Computed tomography is routinely performed at either 6 or 12 months postoperatively, and the stability of the internal structure is determined from the solid bony union that occurs at the hinge site [2, 8, 9]; thereafter, laminoplasty re-closure and the consequent neurological deterioration are unlikely. We hypothesized that a 2-year follow-up might not be essential for patients with symptomatic improvement and solid bony fusion. Nonetheless, as neurological symptoms may change for various reasons, we recommend the implementation of an alternative follow-up system, such as telemedicine.

To date, no study has successfully developed an ML model, based on long-term follow-up data (> 2 years), for the specific optimization of post-cervical laminoplasty outpatient scheduling in patients who had ossification of the posterior longitudinal ligament (OPLL)- or degenerative spinal disease-based cervical stenosis. To address this knowledge gap, in the present study, we enrolled a prospective cohort of patients who underwent cervical laminoplasty (Clinical Trial No. NCT02487901) and were scheduled to undergo follow-up at 1, 3, 6, 12, and 24 months postoperatively [2]. Using data from this prospective cohort, we conducted a pilot study to evaluate the feasibility of using the 12-month postoperative clinical outcomes to predict the 24-month postoperative outcomes. This study was conducted with an aim to develop clinical information-based predictive ML algorithms to stratify patients who would have stable outcomes at the 24-month postoperative timepoint and to identify the most appropriate algorithm for this purpose.

## Methods

### Study design and patient population

This post hoc analysis comprised a subgroup analysis of data from a prospective cohort study of 255 patients who, between July 2015 and April 2017, underwent cervical laminoplasty for OPLL- or degenerative spinal disease-induced cervical stenosis [2]. For all patients, the Arch™ laminoplasty system (DePuy Synthes, Oberdort, Switzerland), with a 12-mm spacer length, was applied during cervical laminoplasty. All surgeries were performed by an experienced surgeon, with experience in conducting more than 500 cervical laminoplasty procedures over a decade. All surgeries were conducted with strict adherence to the standard cervical open laminoplasty procedures. All patients in the cohort were scheduled to visit the clinic at 1, 3, 6, 12, and 24 months postoperatively, at which point clinical outcomes, including the Japanese Orthopedic Association (JOA) scores, were prospectively collected [34]. This secondary analysis comprised data from 80 patients (M: F = 48:32; age, 59.8 ± 10.1 years) who completed a 24-month follow-up schedule. To further validate the robustness and generalizability of our findings, we included a prospective internal test set with 22 additional patients, who underwent data collection between September 2020 and July 2022 and, to ensure consistency in patient selection and data collection, were recruited using the same methods as those used in the original cohort.

**Fig. 1:**
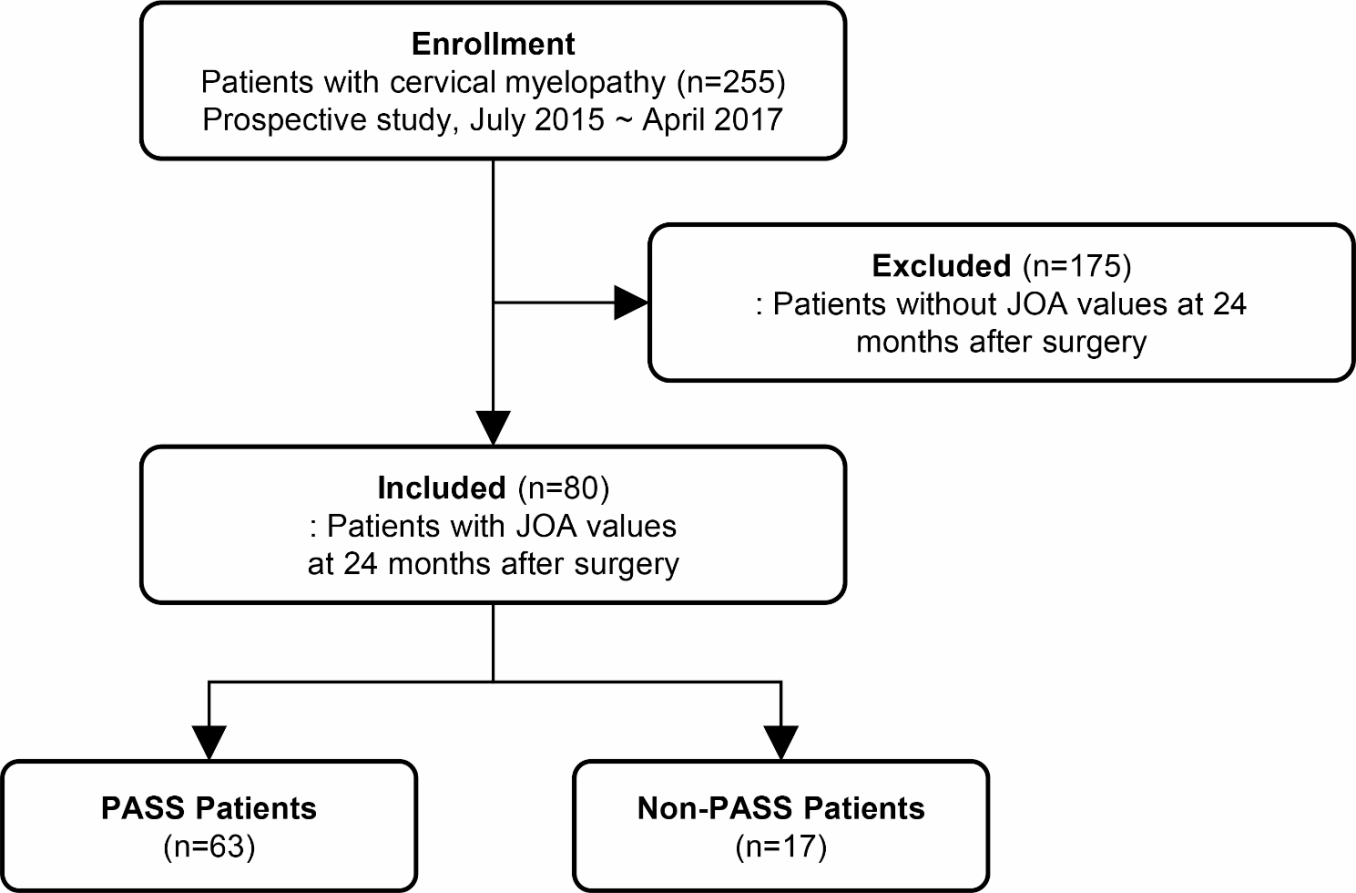
The patients’ flow diagram. The figure shows the overall flow of prospective study. Patients without JOA values at 24 months after surgery were excluded, and all other patients were included. Thus, 255 patients were enrolled in the prospective study, but 175 patients were excluded Abbreviations: JOA: Japanese Orthopedic Association

In this secondary analysis, data from a prospective cohort with cervical myelopathy were used to predict the patient acceptable symptom state (PASS) status at the 24-month postoperative timepoint. Therefore, we excluded patients without the 24-month postoperative JOA data. Consequently, the final study sample comprised 80 of the 255 patients originally enrolled in the cohort. A flow diagram illustrating this process is shown in Fig. 1.

This study conformed to the principles evinced in the Declaration of Helsinki and the Guidelines for Good Clinical Practice. The Ethics Committee and the Institutional Review Board of Seoul National University Hospital approved (approval no. 1505-037-670) the study protocol and, owing to the retrospective nature of this study, waived the requirement of informed consent.

### Variables and ML model implementation

The analysis included 17 clinicoradiological data points as independent variables, including age, sex, body mass index (BMI, kg/m^2^), diabetes, smoking status, occupation, Charlson Comorbidity Index (CCI), preoperative ambulatory status, diagnosis (stenosis vs. myelopathy), presence of high signal intensity on T2-weighted magnetic resonance imaging (HIS), presence of the snake eye sign (SES), ambulatory status at the 1-month postoperative timepoint, preoperative JOA score, and JOA scores at the 1-, 3-, 6-, and 12-month postoperative timepoints (Table 1). The JOA score assesses a patient’s condition in six domains, on a scale from 0 to 17, with higher scores indicating a better patient condition [34]. The PASS was defined as a JOA score ≥ 14.25 at the 24-month postoperative timepoint [35]. Patients who met or did not meet the PASS criteria were coded as 1 and 0, respectively. A PASS score of 1 indicated that the patient was in good condition. These coded values were used as dependent variables, wherein 63 (78.8%) and 17 (21.3%) patients were assigned codes of 1 and 0, respectively. Using the stratified random sampling technique, the training and test sets (8:2) were created; the training set comprised 50 and 14 patients with PASS 1 and 0 and the test set comprised 13 and 3 patients with PASS 1 and 0, respectively. Eight ML algorithms [36] were applied to the variables and dataset, as follows: logistic regression (LR) [37, 38], SVM [39], k-nearest neighbor (kNN) [40], RF [41], extreme gradient boosting (XGBoost) [42], multilayer perceptron (MLP) [43, 44], recurrent neural network (RNN) [45], and long short-term memory (LSTM) [46]. LR is a representative ML algorithm that is broadly applied in various fields. This algorithm predicts a probability between 0 and 1 for PASS, the dependent variable, and is primarily used in binary classification. SVM is an algorithm that maximizes the distance (called a “margin”) between two classes by using a linear kernel function to extend a linear space into a nonlinear space. kNN is one of the simplest available ML algorithms; with new input data, kNN identifies ‘k’ data points close to the existing set and subsequently classifies the data points as the class with the highest frequency of occurrence. We set three as the number of neighbors to consider (*k*). RF is an ensemble technique that combines multiple DTs to improve performance by using a bootstrap aggregation technique that generates a weak classifier for each sample by randomly extracting samples of the same size from the original dataset multiple times. We used 100 trees, the two minimum numbers of samples required to split an internal node, one minimum node size, and the Gini Impurity Index as the node-splitting criteria. XGBoost is an algorithm of the ensemble-boosting technique that inputs errors between the actual and predicted values from previous models as training data and supplements errors using gradients. We used 400 trees, 3 maximum depths, and a 0.1 learning rate. An MLP is a neural network wherein one or more perceptrons form multiple layers, with one or more hidden layers between the input and output layers. The algorithm was developed using stochastic gradient descent (SGD) for weight optimization, and the strength of L2 regularization was set at 1e-5 alpha, with a learning rate of 0.001, 32 neurons in three hidden layers, and a rectified linear unit (ReLU) function. An RNN is defined as an artificial neural network that is characterized by a recurrent structure that processes the inputs and outputs in sequential units. For the RNN model, 32, 10, and 2 hidden state sizes were defined, and the activation function was softmax. Categorical cross-entropy and Adam were used as the loss function and optimizer, respectively. LSTM is an algorithm that was originally developed as one of the RNN algorithms to prevent the vanishing-gradient problem of existing RNNs by using cells, input gates, outputs, and forget gates. The LSTM uses the same hyperparameter settings as the RNN mentioned above. The detailed hyperparameters for each of the eight ML algorithms are listed in Table 2.

**Table 1:** The demographics of patients

	Total (*n* = 80)	PASS = 1 (*n* = 63)	PASS = 0 (*n* = 17)	*p* value
Age (mean, std)	58.76 ± 10.10	57.05 ± 9.38	65.12 ± 10.41	0.008
Sex	Male :48 (60.0%) Female: 32 (40.0%)	Male: 36 (57.1%) Female: 27 (42.9%)	Male: 12 (70.6%) Female: 5 (29.4%)	0.315
BMI (mean, std)	25.78 ± 3.53	26.17 ± 3.42	24.32 ± 3.65	0.072
DM	Yes: 16 (20.0%) No: 64 (80.0%)	Yes: 13 (20.6%) No: 50 (79.4%)	Yes: 3 (17.6%) No: 14 (82.4%)	0.785
Smoking	Yes: 12 (15.0%) No: 68 (85.0%)	Yes :8 (12.7%) No: 55 (87.3%)	Yes: 4 (23.5%) No: 13 (76.5%)	0.267
OA^1^	1:6 (7.5%) 2:19 (23.8%) 3:55 (68.8%)	1:5 (7.9%) 2:15 (23.8%) 3:43 (68.3%)	1:1 (5.9%) 2:4 (23.5%) 3:12 (70.6%)	0.958
CCI (mean, std)	1.80 ± 1.30	1.62 ± 1.18	2.47 ± 1.50	0.042
Preop. Ambulation^2^	1:19 (23.8%) 2:55 (68.8%) 3:6 (7.5%)	1:16 (25.4%) 2:41 (65.1%) 3:6 (9.5%)	1:3( 17.6%) 2:14 (82.4%) 3:0 (0.0%)	0.281
Diagnosis	Stenosis: 3 (3.8%) Myelopathy: 77 (96.3%)	Stenosis: 1 (1.6%) Myelopathy: 62 (98.4%)	Stenosis: 2 (11.8%) Myelopathy: 15 (88.2%)	0.050
HIS	Yes: 77 (96.3%) No: 3 (3.8%)	Yes: 60 (95.2%) No: 3 (4.8%)	Yes: 17 (100.0%) No: 0 (0.0%)	0.359
SES	Yes: 24 (30.0%) No: 56 (70.0%)	Yes: 19 (30.2%) No: 44 (69.8%)	Yes: 5 (29.4%) No: 12 (70.6%)	0.952
Postop. 1 month ambulation^3^	Nulls: 3 (3.8%) 1:28 (35.0%) 2:46 (57.5%) 3:3 (3.8%)	Nulls: 3 (4.8%) 1:23 (36.5%) 2:34 (54.0%) 3:3 (4.8%)	Nulls: 0 (0.0%) 1:5 (29.4%) 2:12 (70.6%) 3:0 (0.0%)	0.484
JOA score preop (mean, std)	11.18 ± 3.28	11.90 ± 2.77	8.47 ± 3.66	0.002
1 month (mean, std)	14.10 ± 2.21	14.60 ± 1.73	12.24 ± 2.80	0.003
3 months (mean, std)	14.92 ± 1.76	15.37 ± 1.38	13.24 ± 2.02	0.001
6 months (mean, std)	15.01 ± 1.75	15.56 ± 1.34	13.00 ± 1.66	0.000
12 months (mean, std)	15.23 ± 1.75	15.83 ± 0.99	13.00 ± 2.15	0.000
24 months (mean, std)	15.14 ± 2.42	16.08 ± 0.81	12.65 ± 1.50	0.000

^1^ Occupational activity; 1: high, 2: intermediate, 3: low

^2^ Preop. Ambulation; 1: fully ambulant, 2: ambulant with an aid, 3: no outdoor self-walking

^3^ Postop. 1 month ambulation; Null: no data, 1: fully ambulant, 2: ambulant with an aid, 3: no outdoor self-walking

Abbreviations: BMI: body mass index; CCI: Charlson comorbidity index; DM: diabetes mellitus; HIS: presence of high signal intensity in T2-weighted magnetic resonance imaging; OA: Occupational Activity; PASS: patient acceptable symptom state; SES: presence of snake eye sign; std: standard deviation

**Table 2:** Hyperparameters of ML algorithms. This table summarizes the key hyperparameters used for various machine learning algorithms in the study. The hyperparameters were tuned and optimized to improve the model performance

Algorithms	Hyperparameter	Value
LR	-	-
SVM	Kernel	Linear
kNN	Number of neighbors (k)	3
RF	Number of trees	100
	Minimum samples to split a node	2
	Minimum node size	1
	Node splitting criterion	Gini Impurity
XGBoost	Number of trees	400
	Maximum depth	3
	Learning rate	0.1
MLP	Optimization algorithm	Stochastic Gradient Descent (SGD)
	L2 regularization strength	1e-5
	Learning rate	0.001
	Number of hidden layers	3
	Number of neurons per hidden layer	32
	Activation function	Rectified Linear Unit (ReLU)
RNN	Hidden state sizes	30, 10, 2
	Activation function	Softmax
	Loss function	Categorical cross-entropy
	Optimizer	Adam
LSTM	Hidden state sizes	30, 10, 2
	Activation function	Softmax
	Loss function	Categorical cross-entropy
	Optimizer	Adam

Abbreviations: ML: machine learning; LR: Logistic regression; SVM: Support vector machines; kNN: k-nearest neighbor; XGBoost: Extreme gradient boosting; RF: Random forests; MLP: Multilayer perceptron; RNN: Recurrent neural network; LSTM: Long short-term memory

### Feature engineering

Patient characteristics, including age, sex, diagnosis, BMI, diabetes, smoking status, and comorbidities, were obtained from the patients’ nursing records. The presence of HIS and SES on T2-weighted magnetic resonance images was assessed, and radiology medical records were referenced. Sex was coded as 1 for male and 0 for female patients, whereas age was recorded as an integer. The diagnosis was coded as 1 and 2, respectively, for stenosis without and with clinical myelopathy, which included an increased deep tendon reflex, positive Hoffmann’s sign, decreased grip and release test, positive Romberg test, or veering on tandem gait. The presence of SES and HIS was coded as 1 if yes and 0 if no. BMI was recorded as a continuous variable. Diabetes was coded as 1 if present, and 0 otherwise. The smoking status was coded as 1 for yes and 0 for no. Occupational classification was undertaken according to the Occupational Activity (OA) criteria established by Steeves et al. [47], with high, intermediate, and low OA coded as 1,2, and 3, respectively. The CCI is a validated method that, based on the presence and severity of 17 specific comorbidities, assigns a weighted score to predict long-term mortality and morbidity [48]. The preoperative and 1-month postoperative ambulatory statuses were coded as 1, 2, and 3 for fully possible ambulation, walking with an aid (stick, walker, etc.), and not being able to walk unaided outside, respectively. Patient prognosis was measured using the JOA pain score (from 0 [worst] to 17 [best]). The dependent variable of PASS status was defined as 1 or 0 if the JOA value at the 24-month postoperative timepoint was ≥ 14.25 and < 14.25, respectively. To eliminate missing values during the 12-month follow-up period, which were imputed using linear interpolation, pre-processing was performed. Missing values for the categorical variables were imputed using a one-hot encoding method. However, patients with missing JOA values the 24-month postoperative timepoint were excluded from the training data to avoid errors in the ground truth.

### Statistical analysis

Table 3 presents a comparison of the excluded and included populations. To assess the potential selection bias, we analyzed intergroup differences in the 17 independent variables. For numerical variables, such as age, BMI, CCI, and JOA scores, we conducted independent sample *t*-tests, which were preceded by Levene’s test for equality of variances to verify the assumption of intergroup homogeneity of variance. When Levene’s test indicated equal or unequal variances (*p* > 0.05 or *p* ≤ 0.05, respectively), a standard *t*-test or Welch’s *t*-test (to adjust for this difference), was performed. For categorical variables, including sex, DM, smoking status, OA, preoperative ambulation, postoperative ambulation, diagnosis, residence, HIS, and SES, a cross-tabulation analysis, followed by a chi-square test, was conducted to determine the intergroup distributional differences. To ensure consistent and robust comparisons between the PASS 1 and 0 groups, the same statistical methods were applied to analyze the variables that have been presented in Table 1.

**Table 3:** Differences in demographics between included and excluded patients

	Total (*n* = 255)	Included (*n* = 80)	Excluded (*n* = 175)	*p* value
Age (mean, std)	59.64 ± 12.14	58.76 ± 10.10	60.03 ± 12.97	0.396
Sex	Male: 181 (71.0%) Female: 74 (29.0%)	Male: 48 (60.0%) Female: 32 (40.0%)	Male: 133 (76.0%) Female: 42 (24.0%)	0.009
BMI (mean, std)	25.37 ± 3.71	25.78 ± 3.53	25.19 ± 3.78	0.226
DM	Yes: 55 (21.6%) No: 200 (78.4%)	Yes: 16 (20.0%) No: 64 (80.0%)	Yes: 39 (22.3%) No: 136 (77.7%)	0.681
Smoking	Yes: 50 (19.6%) No: 205 (80.4%)	Yes: 12 (15.0%) No: 68 (85.0%)	Yes: 38 (21.7%) No: 137 (78.3%)	0.210
OA^1^	1: 20 (7.8%) 2: 44 (17.3%) 3: 191 (74.9%)	1:6 (7.5%) 2:19 (23.8%) 3:55 (68.8%)	1:14 (8.0%) 2:25 (14.3%) 3:136 (77.7%)	0.178
CCI (mean, std)	1.92 ± 1.39	1.80 ± 1.30	1.97 ± 1.44	0.345
Preop. Ambulation^2^	Nulls: 1 (0.4%) 1: 74 (29.0%) 2: 153 (60.0%) 3: 27 (10.6%)	1: 19 (23.8%) 2: 55 (68.8%) 3: 6 (7.5%)	Nulls: 1 (0.6%) 1: 55 (31.4%) 2: 98 (56.0%) 3: 21 (12.0%)	0.162
Diagnosis	Stenosis: 14 (5.5%) Myelopathy: 241 (94.5%)	Stenosis: 3 (3.8%) Myelopathy: 77 (96.3%)	Stenosis: 11 (6.3%) Myelopathy: 164 (93.7%)	0.409
HIS	Yes: 239 (93.7%) No: 16 (6.3%)	Yes: 77 (96.3%) No: 3 (3.8%)	Yes: 162 (92.6%) No: 13 (7.4%)	0.261
SES	Yes: 91 (35.7%) No: 164 (64.3%)	Yes: 24 (30.0%) No: 56 (70.0%)	Yes: 67 (38.3%) No: 108 (61.7%)	0.200
Postop. 1 month ambulation^3^	Nulls: 10 (3.9%) 1: 102 (40.0%) 2: 129 (50.6%) 3: 14 (5.5%)	Nulls: 3 (3.8%) 1: 28 (35.0%) 2: 46 (57.5%) 3: 3 (3.8%)	Nulls: 7 (4.0%) 1: 74 (42.3%) 2: 83 (47.4%) 3: 11 (6.3%)	0.291
JOA score preop (mean, std)	11.10 ± 3.49	11.18 ± 3.28	11.07 ± 3.59	0.816
1 month (mean, std)	13.88 ± 2.60	14.10 ± 2.21	13.78 ± 2.76	0.321
3 months (mean, std)	14.72 ± 2.04	14.92 ± 1.76	14.61 ± 2.19	0.257
6 months (mean, std)	14.87 ± 2.06	15.01 ± 1.75	14.78 ± 2.24	0.408
12 months (mean, std)	15.16 ± 1.96	15.23 ± 1.75	15.10 ± 2.14	0.228
Residence^4^	Nulls: 16 (6.3%) 1: 106 (41.6%) 2: 54 (21.2%) 3: 79 (31.0%)	Nulls: 6 (7.5%) 1: 31 (38.8%) 2: 24 (30.0%) 3: 19 (23.8%)	Nulls: 10 (5.7%) 1: 75 (42.9%) 2: 30 (17.1%) 3: 60 (34.3%)	0.080

^1^ Occupational activity; 1: high, 2: intermediate, 3: low

^2^ Preop. Ambulation; Null: no data, 1: fully ambulant, 2: ambulant with an aid, 3: no outdoor self-walking

^3^ Postop. 1 month ambulation; Null: no data, 1: fully ambulant, 2: ambulant with an aid, 3: no outdoor self-walking

^4^ Residence; Null: no data, 1: live in Seoul, 2: live in Seoul Metropolitan Area except for Seoul (Incheon, Gyeonggi), 3: live outside the Seoul Metropolitan Area

### Evaluation of the ML model

The output of the analysis was the PASS score at the 24-month postoperative timepoint. Eight ML algorithms were applied to the data: LR, SVM, kNN, RF, XGBoost, MLP, RNN, and LSTM. The performance of each algorithm was evaluated from the sensitivity, specificity, positive predictive value (PPV), negative predictive value (NPV), F1-score, accuracy, and area under the receiver operating characteristic curve (AUROC) [49]. To address the issue of a small number of test samples, the k-fold cross-validation method was applied, and the 5-fold cross validation was ultimately used in this study. Additionally, the robustness and generalizability of the model were validated using a prospective internal test set, and the feature importance of the best-performing model was analyzed using Shapley additive explanation (SHAP) values [50].

This study was conducted using Python version 3.6.13, XGBoost version 1.6.1, SciPy version 1.8.1, Scikit-learn version 1.1.1, Seaborn version 0.11.2, Pandas version 1.4.3, NumPy version 1.19.5, TensorFlow version 2.0.0, and Keras version 2.3.1. Statistical analysis was carried out from July 26, 2022, to August 2, 2022.

## Results

### Patient outcomes

The patient demographics are presented in Table 1. Preoperatively, the mean JOA score was 11.18 ± 3.28, which significantly improved to 14.10 ± 2.21 at 1 through 24 months postoperatively (*p* < 0.05, Table 1) and was maintained for 24 months. A significant intergroup difference was observed between PASS 1 and 2 groups at each timepoint (*p* < 0.05, as detailed in Supplementary Material 1).

### Algorithm performance

**Fig. 2:**
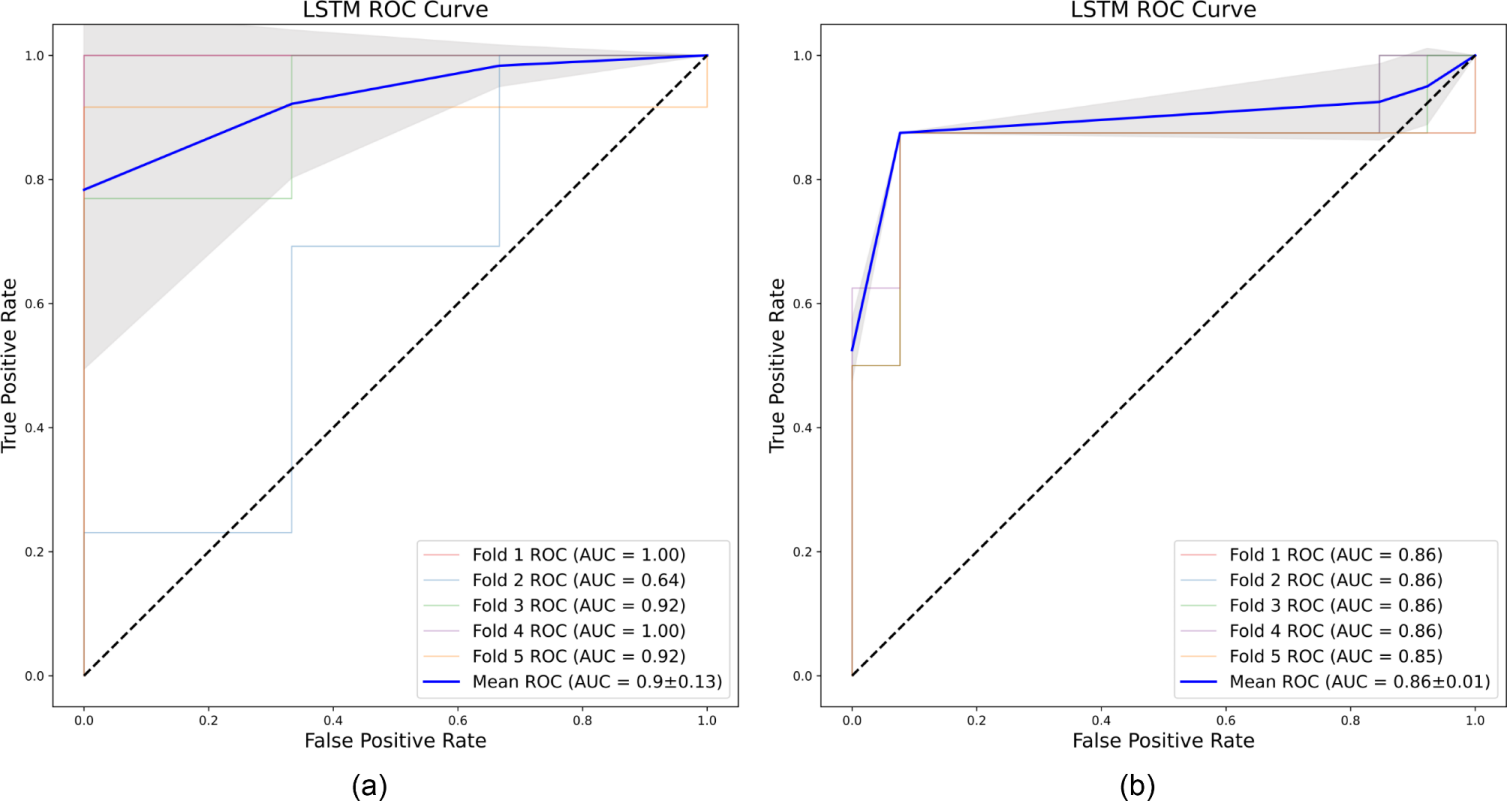
ROC curves of the LSTM-based algorithm. (**a**) ROC curve for the internal test set. (**b**) ROC curve for the prospective internal test set. Due to the 5-fold cross-validation, five ROC curves are generated for each test set, with the mean ROC curve (blue line) representing the average performance of the five models. The shaded area indicates the standard deviation across the folds. Although the AUROC value is lower for the prospective internal test set (0.86 ± 0.01) compared to the internal test set (0.90 ± 0.13), the LSTM-based model still demonstrates strong performance Abbreviations: ROC: Receiver-operating characteristic; AUROC: Area under the receiver operating characteristic; LSTM: Long short-term memory

**Fig. 3:**
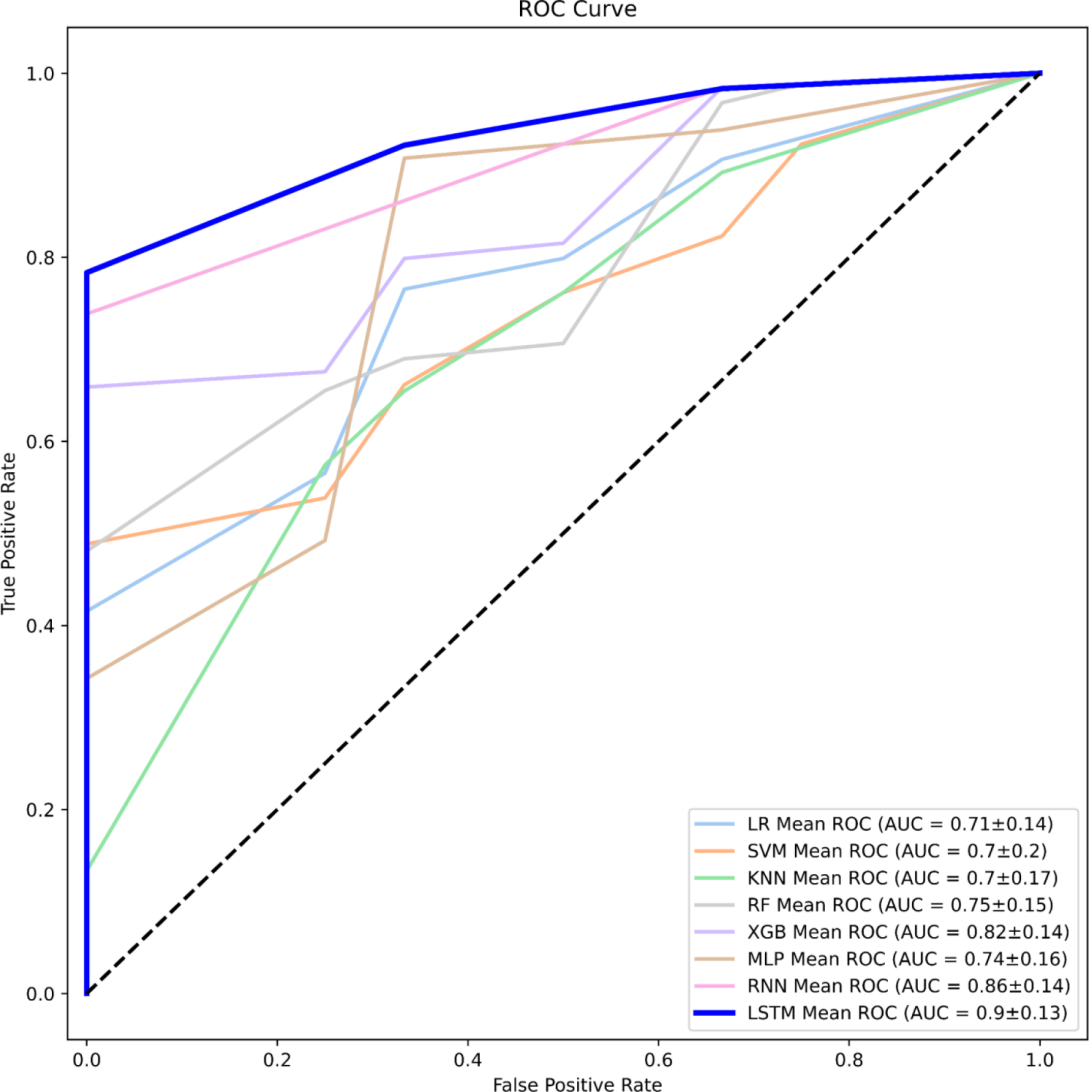
Mean ROC curves for the eight ML models. The plot shows the mean ROC curves for eight ML algorithms: LR, SVM, kNN, RF, XGBoost, MLP, RNN, and LSTM. The AUROC values for each model, along with their standard deviations, are displayed in the legend. The LSTM-based algorithm demonstrates the highest mean AUROC (0.9 ± 0.13), indicating superior performance compared to the other models Abbreviations: ROC: Receiver-operating characteristic; AUROC: Area under the receiver operating characteristic; LR: Logistic regression; SVM: Support vector machines; kNN: k-nearest neighbor; XGBoost: Extreme gradient boosting; RF: Random forests; MLP: Multilayer perceptron; RNN: Recurrent neural network; LSTM: Long short-term memory; ML: Machine learning

**Fig. 4:**
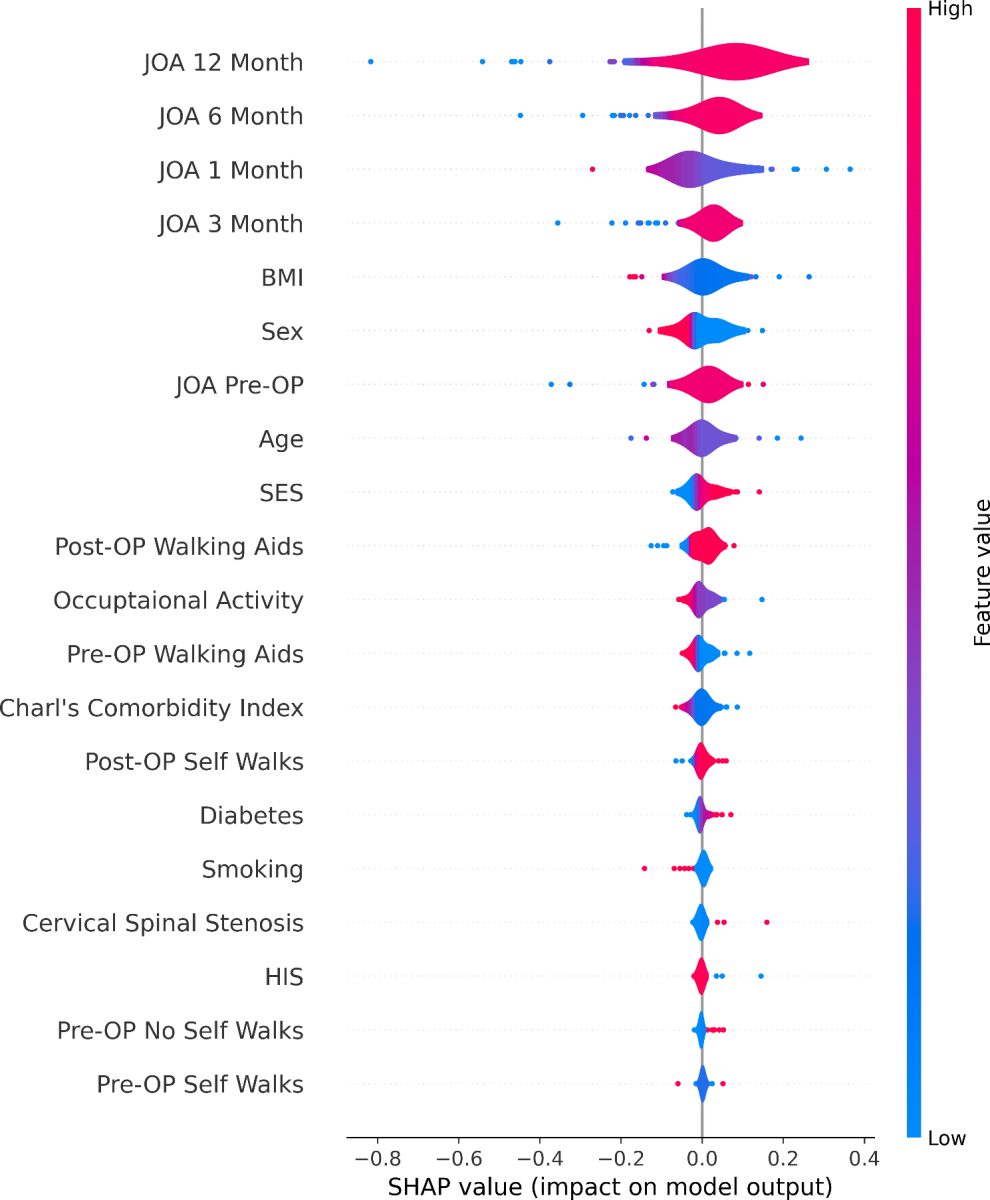
Shapley additive explanation (SHAP) values of all 17 independent variables in the top-performing LSTM-based algorithm Abbreviations: SHAP: Shapley additive explanation; LSTM: Long short-term memory

The performances of the eight ML algorithms are presented in Table 4. For each evaluation metric, the performance was analyzed five times using 5-fold cross validation. In terms of the average values, the algorithm with the best performance for each metric, based on the specificity, sensitivity, PPV, NPV, F1-score, accuracy, and AUROC, was LSTM (specificity, 0.883 ± 0.211; sensitivity, 0.967 ± 0.041; PPV, 0.955 ± 0.061; NPV, 0.893 ± 0.137; F1-score, 0.960 ± 0.0.044; accuracy, 0.938 ± 0.069; and AUROC, 0.900 ± 0.130114). A temporal external test of the LSTM-based algorithm was conducted on the test set comprising 22 patients. This model was selected because of its excellent performance and generated the following results from the prospective internal test set: specificity, 0.892 ± 0.062; sensitivity, 0.875 ± 0.0; PPV, 0.84 ± 0.07; NPV, 0.92 ± 0.006; F1-score, 0.856 ± 0.039; accuracy, 0.886 ± 0.038; and AUROC, 0.858 ± 0.007. Figure 2 shows the receiver operating characteristic (ROC) curves for the LSTM-based algorithm, where (a) represents the ROC curve for the internal test set and (b) represents the ROC curve for the prospective internal test set. Figure 3 shows the ROC curves of the eight ML models. The threshold for all ROC curves was set to 0.5. This figure provides a visual comparison of the performance of each model for distinguishing between patients with stable outcomes and those who require further follow-up. For more detailed information, refer to Figs. S1–S7. Figure 4 shows the feature importance of all 17 independent variables in the top-performing LSTM-based algorithm, as determined from the SHAP values.

**Table 4:** The performance of the eight ML algorithms

ML Model	Sensitivity	Specificity	PPV	NPV	F1-Score	Accuracy	AUROC
LR	0.91 ± 0.03	0.52 ± 0.17	0.88 ± 0.03	0.58 ± 0.15	0.89 ± 0.03	0.83 ± 0.05	0.71 ± 0.09
SVM	0.78 ± 0.15	0.65 ± 0.23	0.90 ± 0.06	0.49 ± 0.11	0.82 ± 0.08	0.75 ± 0.08	0.71 ± 0.05
kNN	0.94 ± 0.06	0.47 ± 0.25	0.87 ± 0.05	0.67 ± 0.37	0.90 ± 0.04	0.84 ± 0.08	0.70 ± 0.14
RF	0.92 ± 0.12	0.58 ± 0.33	0.90 ± 0.06	0.62 ± 0.38	0.90 ± 0.05	0.85 ± 0.06	0.75 ± 0.13
XGBoost	0.94 ± 0.06	0.70 ± 0.40	0.93 ± 0.08	0.63 ± 0.34	0.93 ± 0.02	0.89 ± 0.05	0.82 ± 0.18
MLP	0.95 ± 0.06	0.53 ± 0.20	0.88 ± 0.05	0.80 ± 0.27	0.92 ± 0.04	0.86 ± 0.07	0.74 ± 0.12
RNN	0.95 ± 0.04	0.77 ± 0.29	0.94 ± 0.07	0.79 ± 0.19	0.95 ± 0.05	0.91 ± 0.09	0.86 ± 0.16
LSTM	0.97 ± 0.04	0.88 ± 0.21	0.96 ± 0.06	0.89 ± 0.14	0.96 ± 0.04	0.94 ± 0.07	0.90 ± 0.13

Abbreviations: AUROC: area under the receiver operating characteristic; ML: machine learning; NPV: negative predictive value; PPV: positive predictive value; LR: Logistic regression; SVM: Support vector machines; kNN: k-nearest neighbor; XGBoost: Extreme gradient boosting; RF: Random forests; MLP: Multilayer perceptron; RNN: Recurrent neural network; LSTM: Long short-term memory

## Discussion

This study aimed to evaluate the predictability of the 2-year postoperative outcomes based on the 1-year postoperative outcomes. To achieve this, various ML algorithms were tested, with the LSTM exhibiting the best average performance. The high specificity (0.883 ± 0.211) of this LSTM-based algorithm indicates that it could accurately identify patients who would need to visit the outpatient clinic (i.e., patients with PASS = 0) at the 24-month postoperative timepoint. Despite using a relatively small training set, the algorithm demonstrated strong performance on a prospective internal test set, and this supports its generalizability. These results may be useful for modifying the post-cervical laminoplasty clinical follow-up schedule.

### Inter-model comparison

In this study, we tested five classical ML algorithms (LR, SVM, kNN, RF, and XGBoost) as well as three deep-learning algorithms (MLP, RNN, and LSTM), of which the RNN- and LSTM-based algorithms demonstrated a higher average performance across all metrics than the classical ML algorithms. The superior performance of time series-based deep-learning models, such as LSTM and RNN, could be attributed to their ability to effectively process sequential data [51, 52]. In this context, the JOA values were ordered chronologically, indicating that past patient outcomes significantly influenced future outcomes, and thereby makes these models particularly well-suited for this task.

As shown in Fig. 3, the ROC curve for the LSTM-based algorithm was skewed more toward the top-left corner than in any of the other models, which resulted in an AUROC value > 0.9 that indicates excellent performance. In the present study, the high specificity (0.883 ± 0.211) of the model was considered particularly important as it accurately identifies patients who need to visit an outpatient clinic at 2 years postoperatively (i.e., those with PASS 0). Therefore, we concluded that the LSTM-based model was the most suitable for this purpose.

### Feature importance

By calculating the impact of each feature on the predicted outcome, the SHAP values for the LSTM-based algorithm (Fig. 4) provide insights into how each feature contributes to the model’s predictions. The top 10 features, ranked by SHAP values, include the JOA scores at the 12-, 6-, 1-, and 3-month postoperative timepoints; BMI; preoperative JOA score; sex; age; SES; and preoperative ambulatory status. Notably, BMI exhibited a negative correlation with predicted outcomes, whereas sex showed a negative correlation for male patients and a positive correlation for female patients. Specifically, the JOA score at the 1-month postoperative timepoint negatively correlated, whereas the JOA scores at the 3-, 6-, and 12-month postoperative timepoints positively correlated, with the model predictions. This underscores the significance of time-series data up to the 12-month postoperative timepoint for predicting the 2-year postoperative outcomes. Detailed SHAP values for each fold are shown in Figs. S8–S12.

The high SHAP values of the JOA scores indicate that these sequential clinical outcomes are crucial for determining whether a patient will achieve satisfactory results at the 24-month postoperative timepoint. This result aligns with the ability of the LSTM-based algorithm to effectively capture the temporal progression of patient outcomes. Furthermore, clinical information such as age and SES, contributed significantly, and this underscores the model’s capacity to integrate both time-dependent and static variables for accurate prediction.

This detailed analysis confirmed the clinical relevance of the features selected for the LSTM-based algorithm, and thereby enable more informed clinical decision-making by using both time-series data and clinical information.

### Alternative approaches in resource-limited settings

If a hospital environment lacks the computational resources required to deploy an LSTM-based algorithm for inference, a classical ML model may be a suitable alternative. In this case, the XGBoost model is recommended as the next-best option because it demonstrates the highest specificity among the five classical ML models.

Additionally, it may be challenging to collect all the 17 clinicoradiological values introduced in this study for all outpatients in a hospital setting. In such cases, variables with the highest SHAP values (e.g., JOA scores up to 12 months postoperatively), as shown in Fig. 4, should be prioritized for data collection.

### Clinical application

In patients who underwent cervical laminoplasty, the patient’s condition stabilized following solid bony fusion at the hinge of the reflected lamina, which occurred between 6 and 12 months [2, 8, 9, 32]. Therefore, if a patient’s condition stabilizes for a year, it is unlikely to worsen subsequently [2, 8, 9]. These results indicate that patients who achieve stable clinical improvement may not need to visit outpatient clinics frequently. Given these findings, it would be practical to develop an LSTM-based algorithm that uses the clinicoradiological data from the first postoperative year to identify patients who may safely forego the 2-year postoperative follow-up clinic visit. The identification of patients who meet the PASS criteria could reduce the need for routine in-person follow-up visits and thereby optimize the use of limited medical resources. The algorithm developed in this study could be integrated into an electronic medical information system to assist decision-making for clinical follow-up scheduling. However, this algorithm is not perfect, and this may have led to patients missing clinic visits despite worsening clinical outcomes [1–8, 10–12, 32]. Therefore, this algorithm should not be used to completely exempt patients from clinical visits. Instead, it can be employed to modify the visit frequency or guide the incorporation of alternative follow-up methods, such as telemedicine [53–55]. However, it is crucial to note that, although telemedicine offers a viable option for reducing the number of in-person visits, particularly for patients with a PASS 1, to ensure that patient safety is prioritized, it should be considered an adjunct, rather than a replacement, of direct clinical assessment.

### Research significance and novelty

Our study builds on prior research that highlights the importance of predicting patient no-shows and optimizing outpatient schedules, based on patient characteristics, and minimizing unnecessary clinic visits to efficiently use medical resources [13, 15–19, 56]. Importantly, this novel study applied an ML-based approach to optimize outpatient follow-up schedules, specifically for patients with cervical myelopathy after cervical laminoplasty, with follow-up > 2 years. Although ML models have been previously applied to optimize outpatient scheduling for other patient populations, such as those with lumbar disc herniation [29], no study had specifically developed post-laminoplasty schedules for patients with cervical myelopathy by using long-term (> 2-year) follow-up data. This highlights the uniqueness and significance of our study in addressing this clinical need.

In addition, similar to prior research that demonstrated temporal generalization using prospective internal test sets in different periods [57–59], our LSTM-based algorithm successfully underwent temporal generalization validation using a prospective internal test set. As shown in Fig. 2, although the ROC curve for the prospective internal test set exhibited a slightly lower AUROC value compared to the internal test set, its specificity (0.892 ± 0.062) was higher than that of the internal test set (0.883 ± 0.211) and indicated that our model is not overfitted to a specific cohort, but rather, demonstrates generalizability across different periods.

### Variables utilized for ML

The current study used the JOA score as the most important clinical outcome measure in patients with cervical myelopathy. Although the JOA is widely used to measure the post-cervical laminoplasty clinical outcome, it may not sufficiently represent a patient’s status. The inclusion of additional variables, such as quality of life and numeric rating pain scores for the neck and arm, would therefore help to improve the performance of the algorithm. Defining the PASS by using multiple variables may further improve the reliability of the ML algorithm; however, this proposal was not explored in the present study, and should be further investigated in future studies.

### Limitations

This study had some limitations. First, a small number of data points were used for the training set. Besides the limited sample size, the study population was drawn exclusively from a single institution – Seoul National University Hospital – that primarily serves Korean patients, and this resulted in a lack of ethnic diversity. The data collection period was limited, which may have restricted the generalizability of the findings. Although clinical information was collected prospectively from 255 patients, owing to missing datapoints, data from only 80 patients were usable. A five-fold cross-validation algorithm was applied to address the issue of the small sample. Furthermore, to demonstrate its generalizability, the algorithm was validated using a prospective internal test set that was collected at a significantly different period, which highlights its robustness across temporal variations. However, before general clinical use, the algorithm should be tailored and optimized by analyzing large datasets. Further research using large and complete datasets from various institutions, and including patients of diverse ethnic background, is necessary to optimize the algorithm. A prospective cohort study to evaluate the clinical utility and generalizability of this model is required. Second, the PASS criteria of the JOA may not fully represent the patient’s status. For example, depending only on the clinical outcomes, OPLL progression may be missed. Further research is required to address this issue and to accurately identify patients who may not require in-person clinic visits. Despite these limitations, this study is the first to apply ML algorithms to predict the post-cervical laminoplasty 24-month postoperative outcomes using data from a prospective cohort. This information could enable the development of algorithms to modify the clinic-visit schedule or the type of clinic.

## Conclusions

Despite the small sample size, using clinicoradiological data from a prospective cohort, this study demonstrated the robust predictive performance of an ML algorithm for the 24-month postoperative outcome. Based on their clinical data for up to 1 year, the predictive identification of patients who would achieve stable outcomes at the 24-month postoperative timepoint could facilitate more efficient medical resource utilization.

## Electronic supplementary material

Below is the link to the electronic supplementary material.

Supplementary Material 1

Supplementary Material 2

## Data Availability

The materials and data used in this study will be shared upon reasonable written request to the corresponding author. Access to the code requires the data access agreement and permission from the institutional review board.

## Abbreviations

AUROC: Area under the receiver operating characteristic
BMI: Body mass index
CCI: Charlson Comorbidity Index
DM: Diabetes mellitus
HIS: Presence of high signal intensity in T2-weighted magnetic resonance imaging
ML: Machine learning
NPV: Negative predictive value
OA: Occupational Activity
PASS: Patient acceptable symptom state
PPV: Positive predictive value
SES: Presence of snake eye sign
Std: Standard deviation
