# Supplementary material for: Machine-learning-based models for the optimization of post-cervical spinal laminoplasty outpatient follow-up schedules
Source: BMC Med Inform Decis Mak. 2024 Sep 30;24:278. doi: 10.1186/s12911-024-02693-y (PMC11440713; doi:10.1186/s12911-024-02693-y)
Supplement: Supplementary file 1 — Supplementary Material 1 [file 12911_2024_2693_MOESM1_ESM.docx]

**1. Supplemental Discussions**

**1.1 Selection Bias**

Patients were excluded from the analysis due to missing data. The p-value between the excluded and included populations is greater than 0.05 in all independent variables except for sex (Table 2). Therefore, selection bias is negligibly small for 16 variables. There is a possibility that selection bias remains for the sex parameter. However, there is little possibility that the model is overfitted because there is conceivably no bias in neurological variables more critical to the patient's prognosis, such as JOA variables.

Also, the demographic data was not a reason for exclusion from this study. All patients were ambulatory and followed up in the outpatient clinic in a prospective study; fortunately, there was no neurological complication. No patients were in a nursing facility. In addition, Table 2 shows the results of analyzing the residence among the patient's demographic data. The patient's residential area was classified by the distance from Seoul National University Hospital, and in close order, it was divided into Seoul, Metropolitan Area except for Seoul (Incheon, Gyeonggi), outside Seoul Metropolitan Area. As a result of comparing the distribution of the residential area between the included and excluded populations, the p-value is more than 0.05, so the selection bias due to the residential area is slight.

Therefore, selection bias is negligible in applying the exclusion criteria to the prospective population, and it is less likely that the ML model was overfitted to the included population.
