# Supplementary material for: Machine-learning-based models for the optimization of post-cervical spinal laminoplasty outpatient follow-up schedules
Source: BMC Med Inform Decis Mak. 2024 Sep 30;24:278. doi: 10.1186/s12911-024-02693-y (PMC11440713; doi:10.1186/s12911-024-02693-y)
Supplement: Supplementary file 2 — Supplementary Material 2 [file 12911_2024_2693_MOESM2_ESM.docx]

**2. Supplemental figures**

**2.1 ROC Curves for Each ML Model**


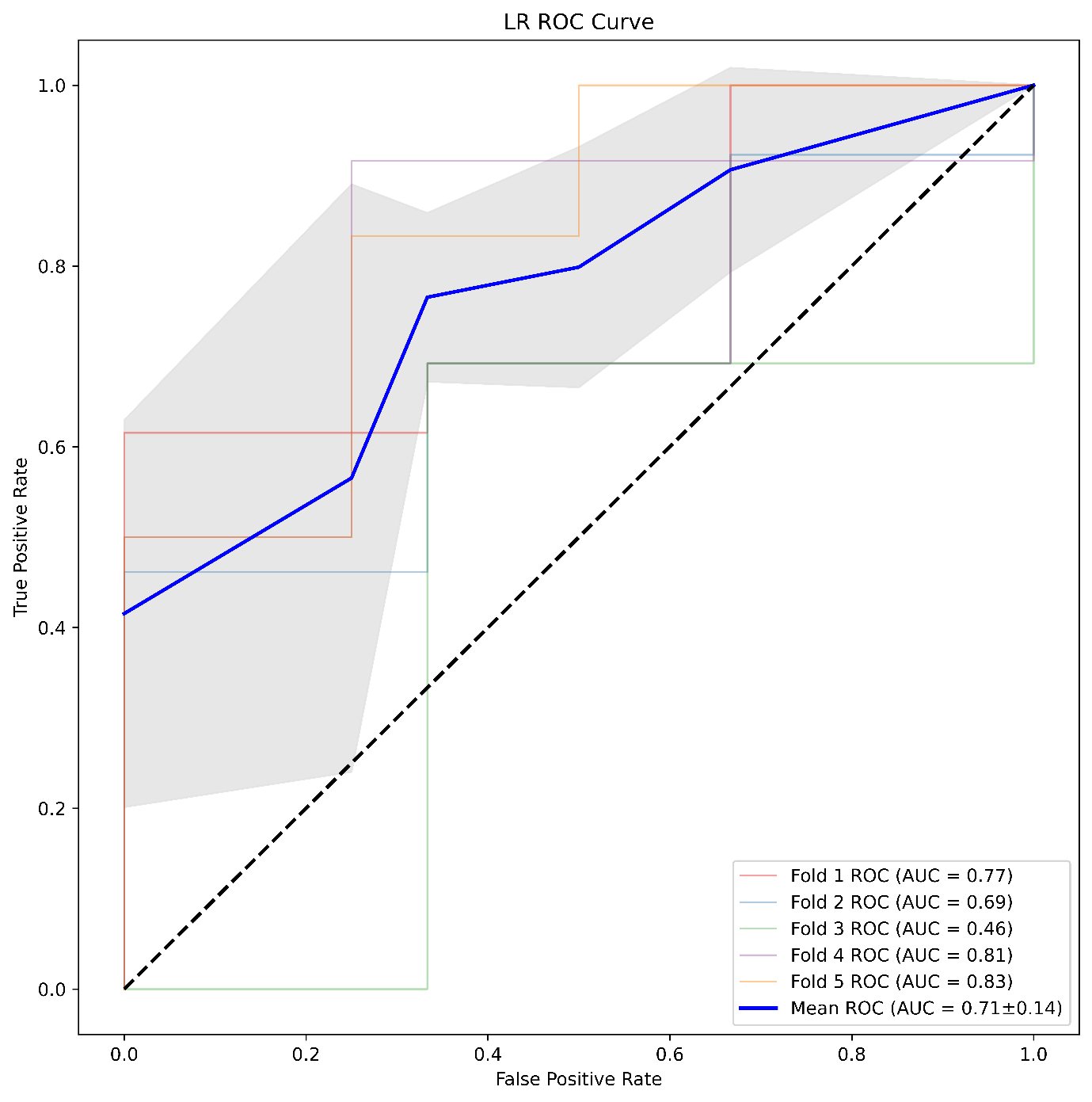


**Figure S1. ROC curve for the LR-based algorithm.** The plot shows the ROC curves for each of the five cross-validation folds (Fold 1 to Fold 5), along with their respective AUC values. The blue line represents the ROC curve of the mean model, calculated across all folds, with the shaded area indicating the standard deviation.


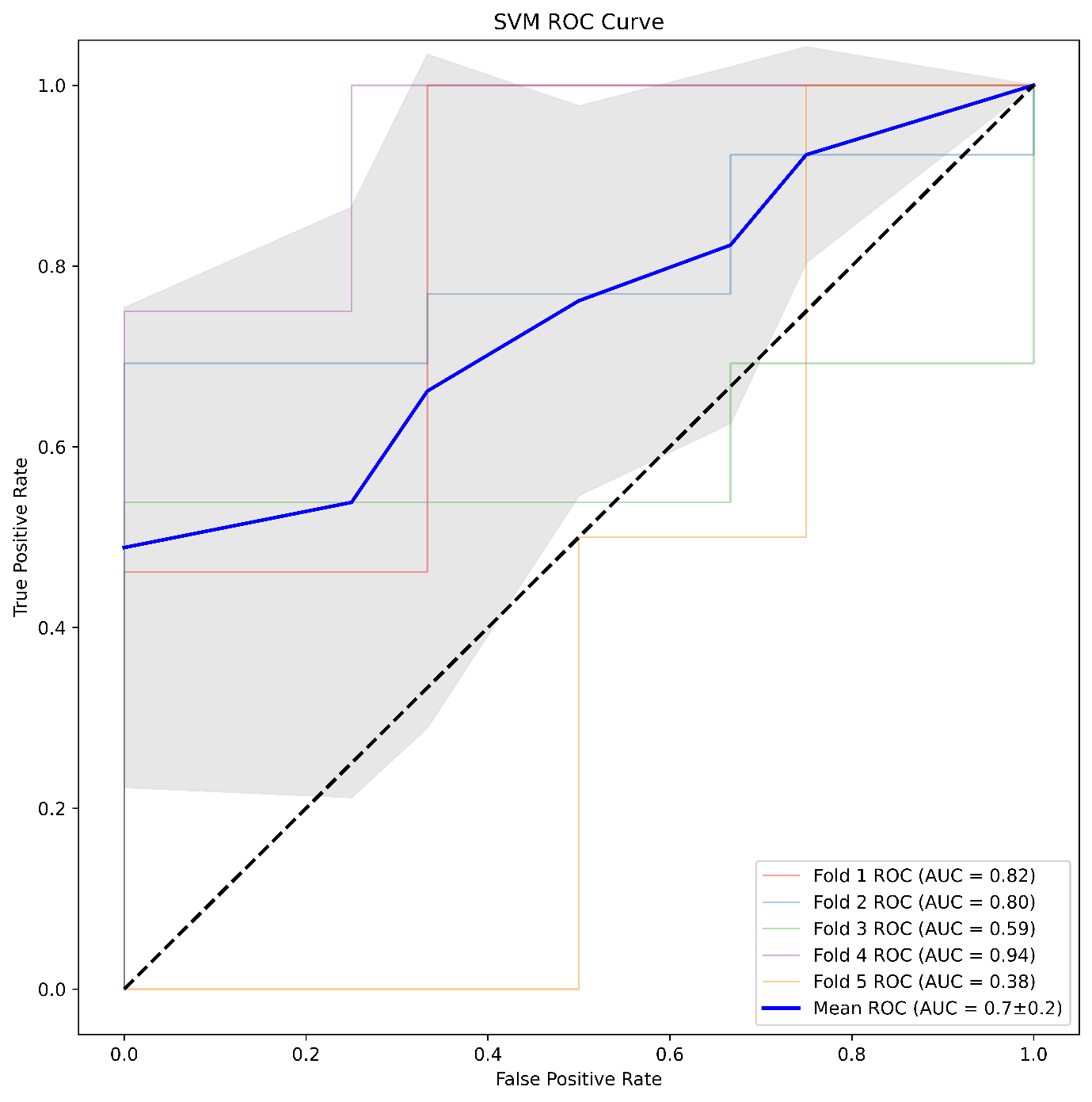


**Figure S2. ROC curve for the SVM-based algorithm.** The plot shows the ROC curves for each of the five cross-validation folds (Fold 1 to Fold 5), along with their respective AUC values. The blue line represents the ROC curve of the mean model, calculated across all folds, with the shaded area indicating the standard deviation.


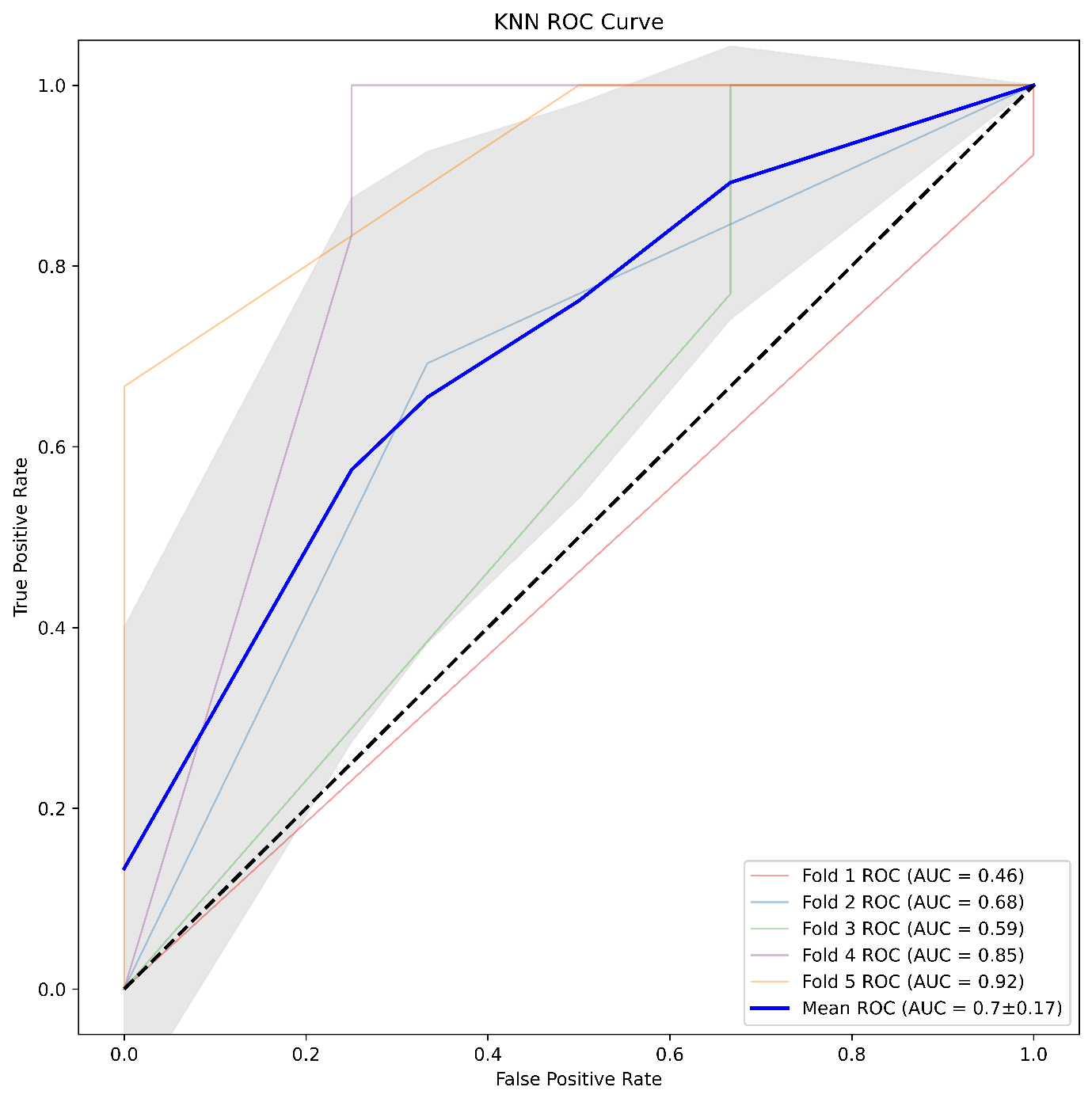


**Figure S3. ROC curve for the kNN-based algorithm.** The plot shows the ROC curves for each of the five cross-validation folds (Fold 1 to Fold 5), along with their respective AUC values. The blue line represents the ROC curve of the mean model, calculated across all folds, with the shaded area indicating the standard deviation.


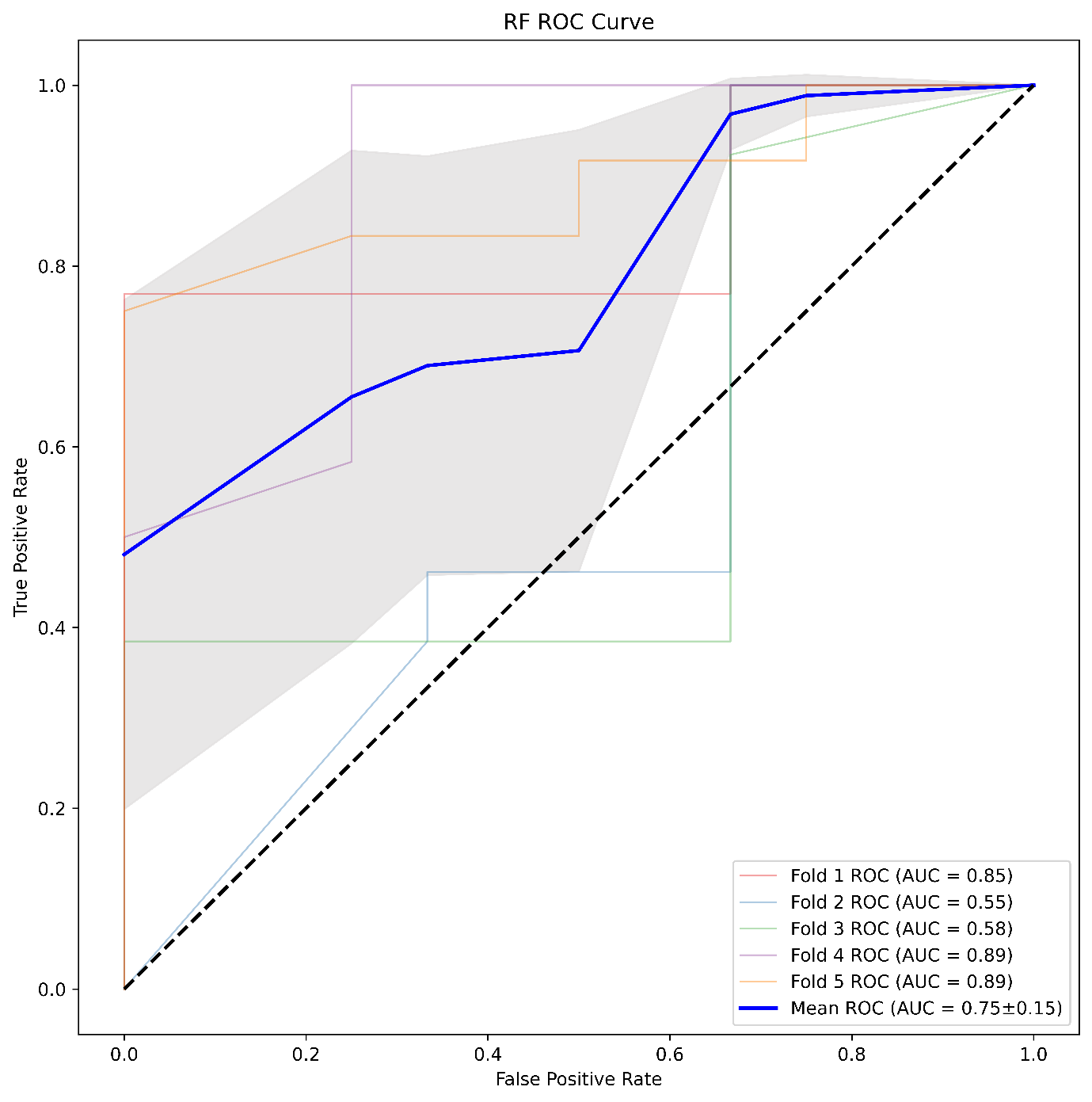


**Figure S4. ROC curve for the RF-based algorithm.** The plot shows the ROC curves for each of the five cross-validation folds (Fold 1 to Fold 5), along with their respective AUC values. The blue line represents the ROC curve of the mean model, calculated across all folds, with the shaded area indicating the standard deviation.


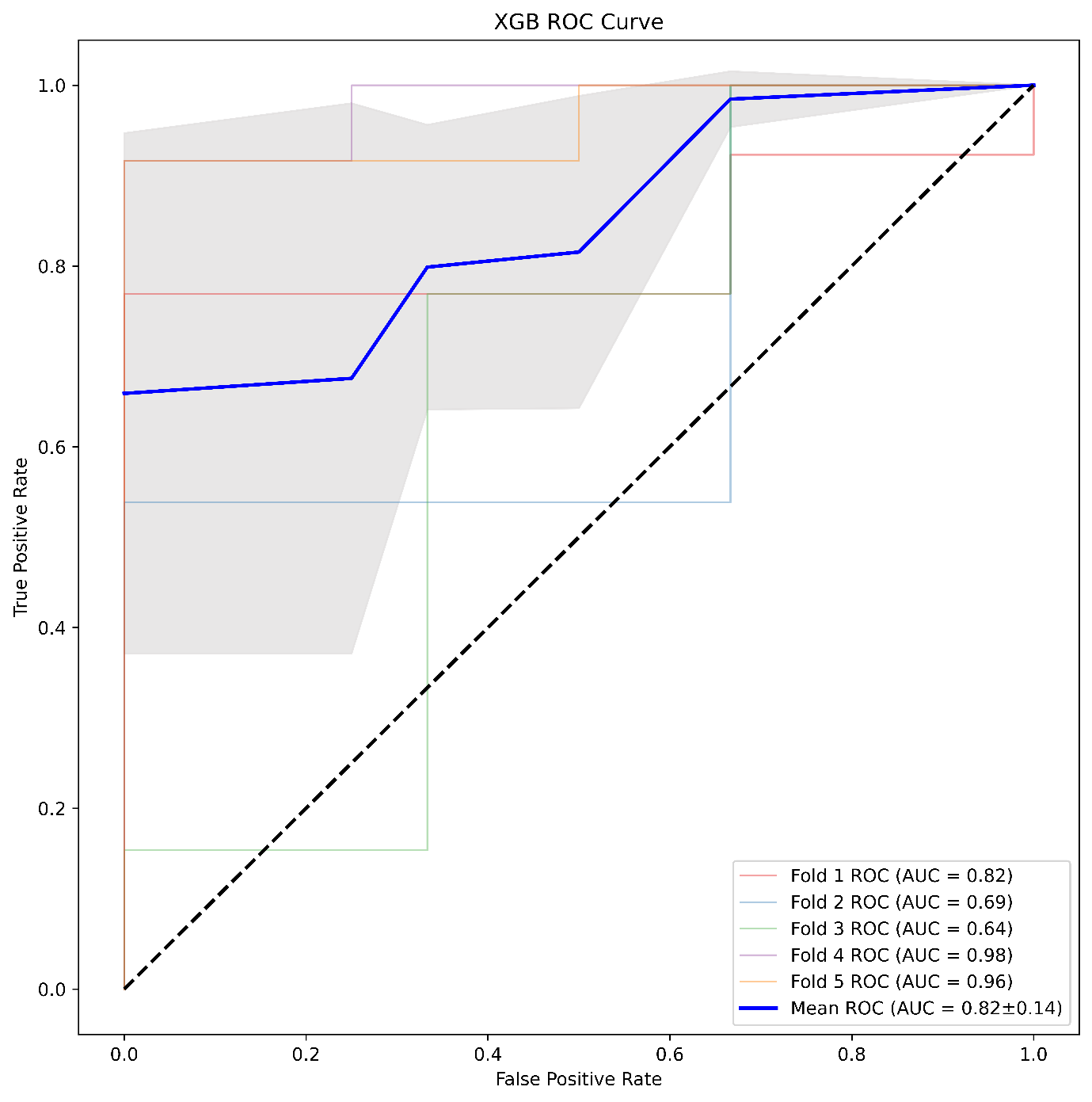


**Figure S5. ROC curve for the XGBoost-based algorithm.** The plot shows the ROC curves for each of the five cross-validation folds (Fold 1 to Fold 5), along with their respective AUC values. The blue line represents the ROC curve of the mean model, calculated across all folds, with the shaded area indicating the standard deviation.


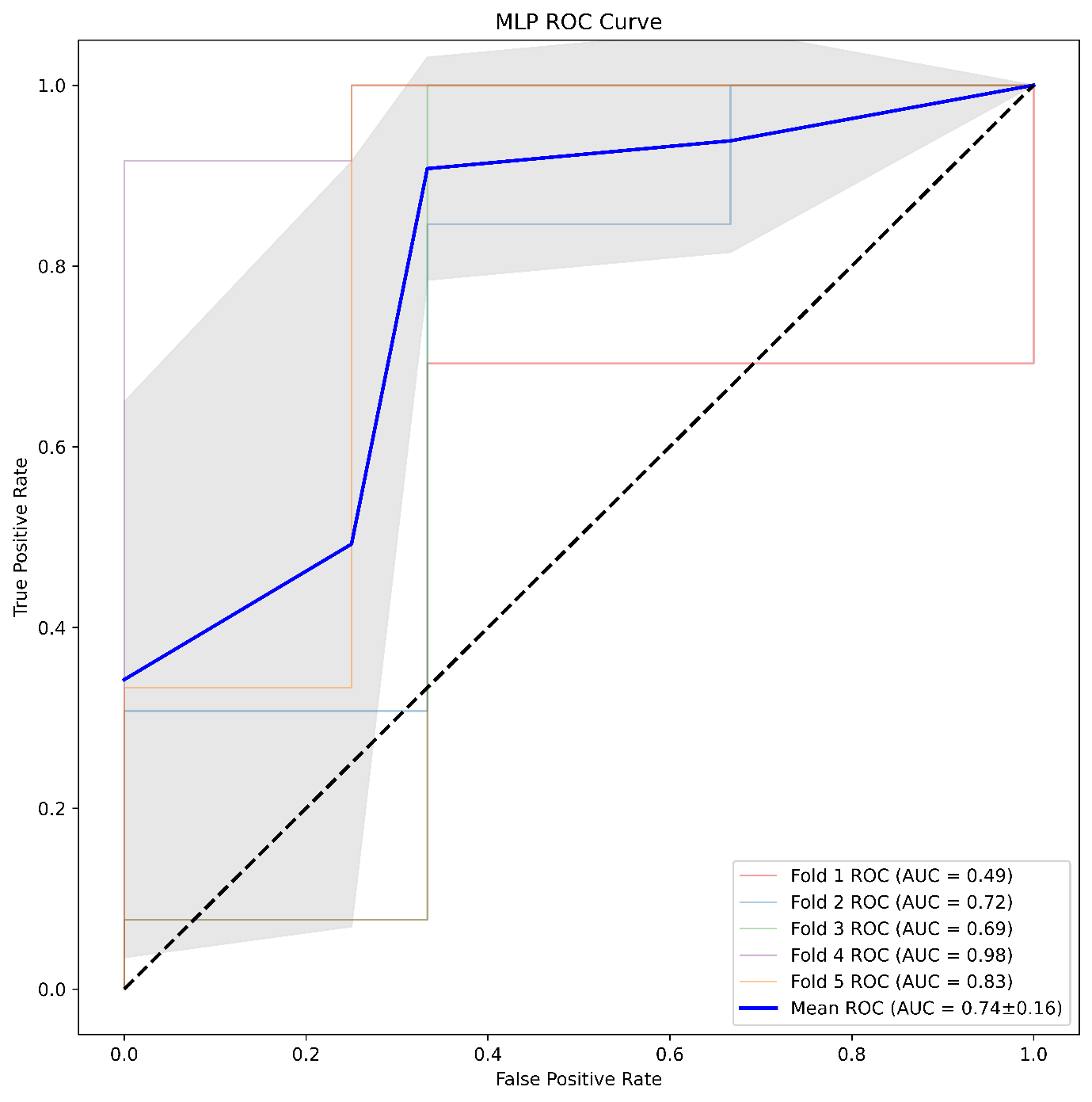


**Figure S6. ROC curve for the MLP-based algorithm.** The plot shows the ROC curves for each of the five cross-validation folds (Fold 1 to Fold 5), along with their respective AUC values. The blue line represents the ROC curve of the mean model, calculated across all folds, with the shaded area indicating the standard deviation.


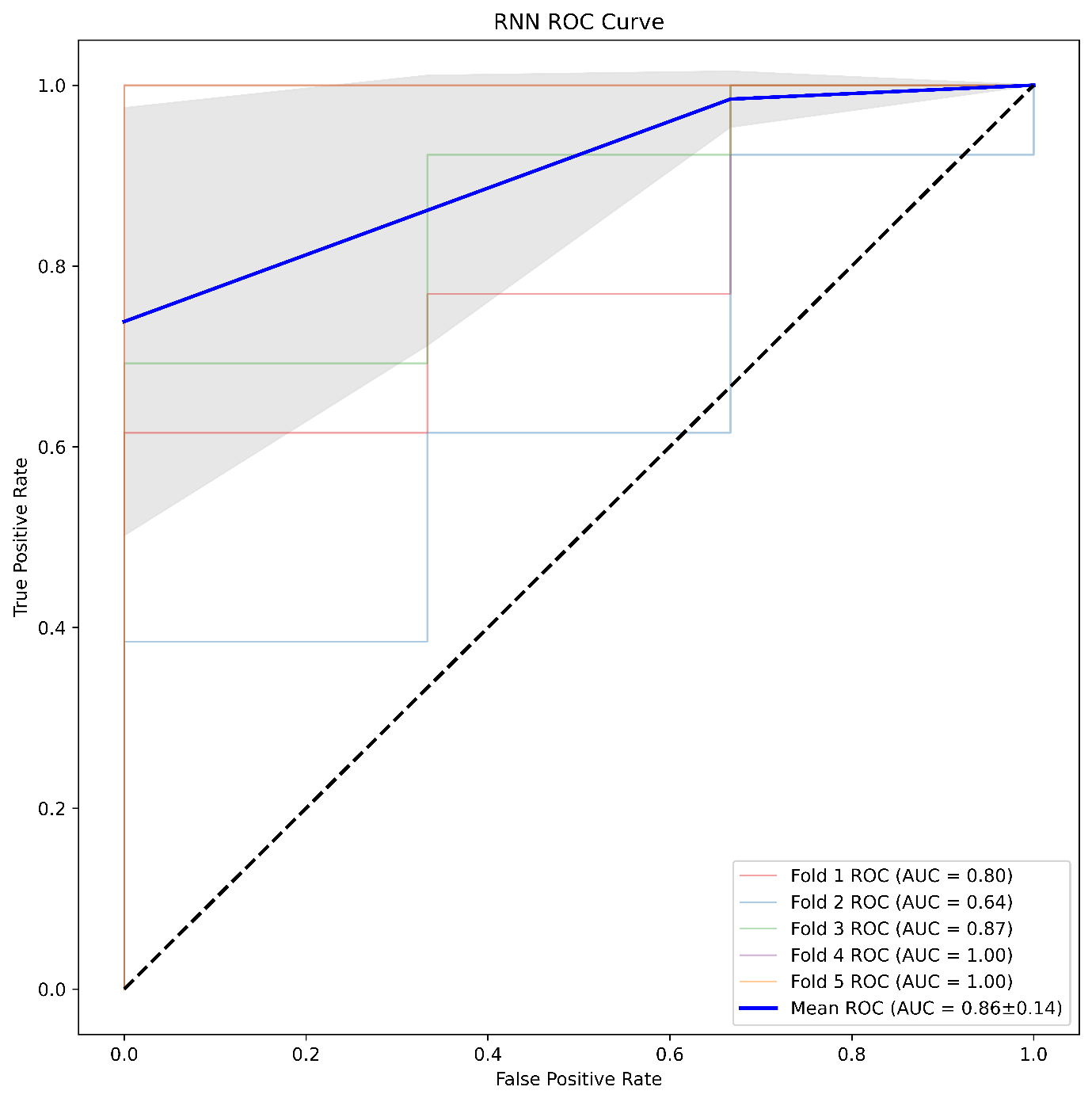


**Figure S7. ROC curve for the RNN-based algorithm.** The plot shows the ROC curves for each of the five cross-validation folds (Fold 1 to Fold 5), along with their respective AUC values. The blue line represents the ROC curve of the mean model, calculated across all folds, with the shaded area indicating the standard deviation.

**2.2 SHAP Values for Each Fold of the LSTM-based Model**


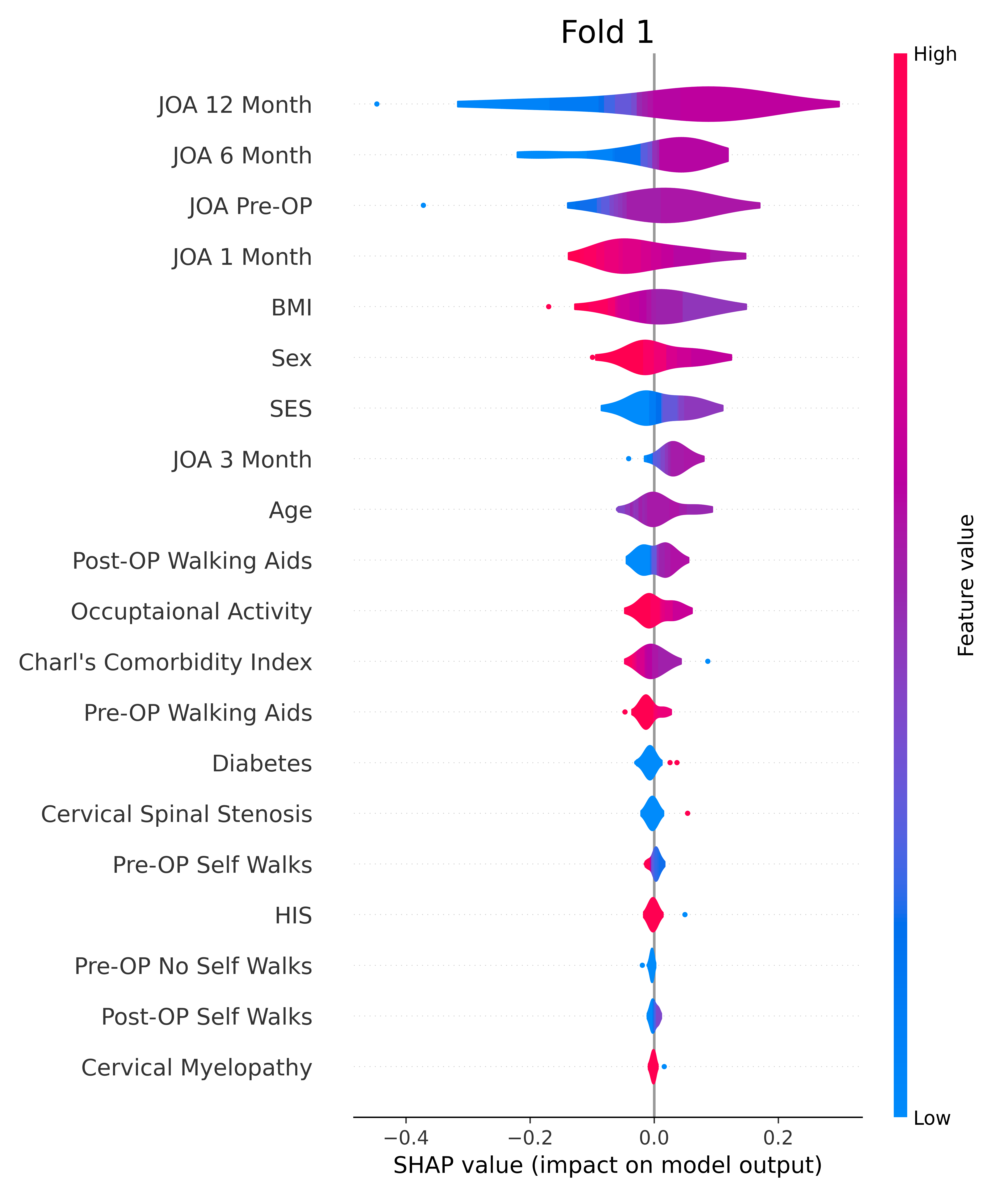


**Figure S8.** **Shapley Additive Explanation (SHAP) values for the LSTM-based model in Fold 1.** The most important feature is the JOA score at 12 months, followed by the JOA score at 6 months, the preoperative JOA score, and the JOA score at 1 month. BMI is also a significant contributor, with sex, SES, and the JOA score at 3 months being important variables. Age and occupational activity round out the top 10 features, demonstrating the importance of both clinical and demographic variables in predicting postoperative outcomes.


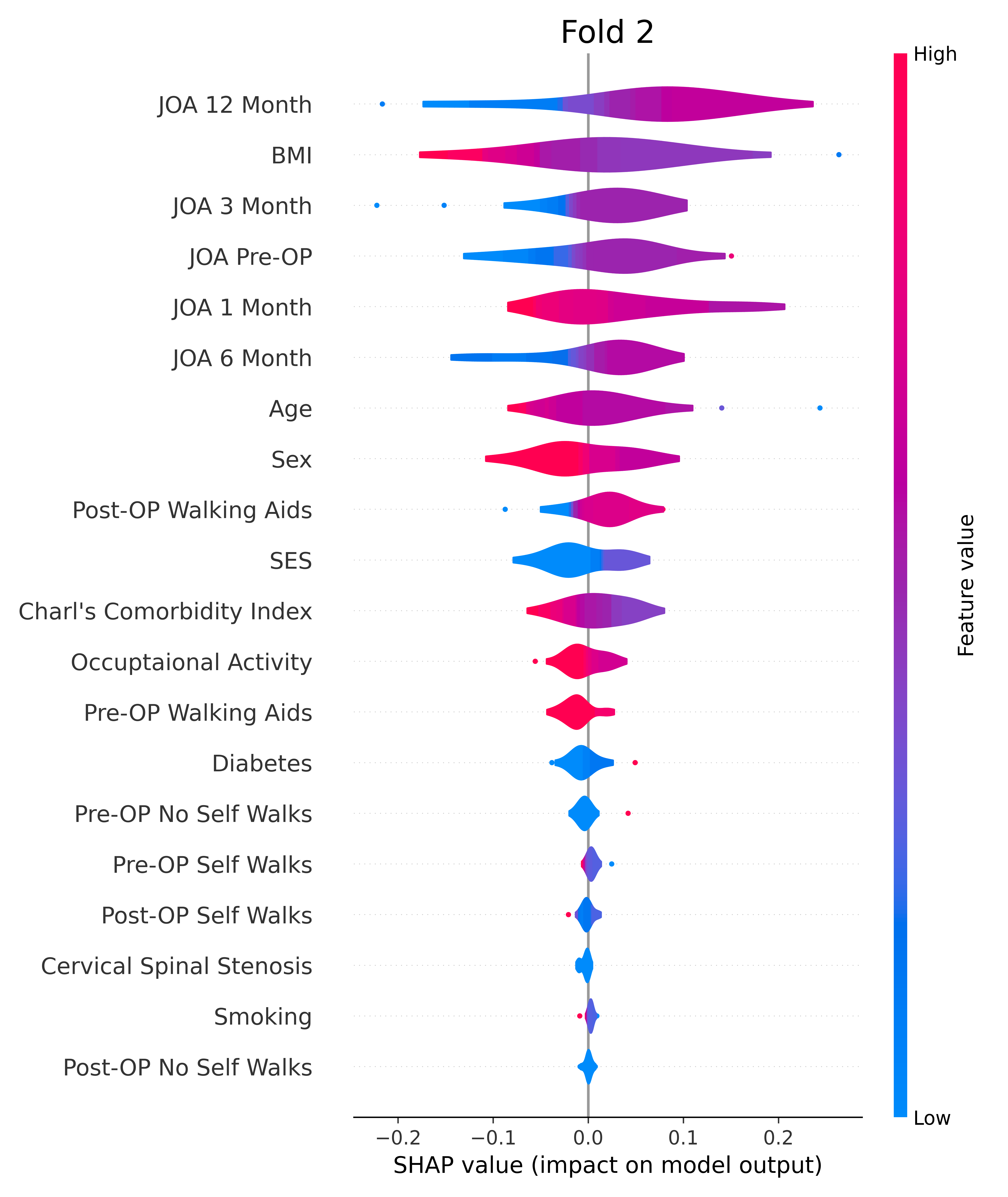


**Figure S9.** **Shapley Additive Explanation (SHAP) values for the LSTM-based model in Fold 2.** The most important feature is the JOA score at 12 months, followed by BMI, the JOA score at 3 months, and the preoperative JOA score. The JOA score at 1 month and JOA score at 6 months also significantly contribute to the predictions, along with sex and age. Post-operative walking aids and SES round out the top 10 features, highlighting the combined importance of clinical outcomes and patient-specific factors in predicting postoperative results.


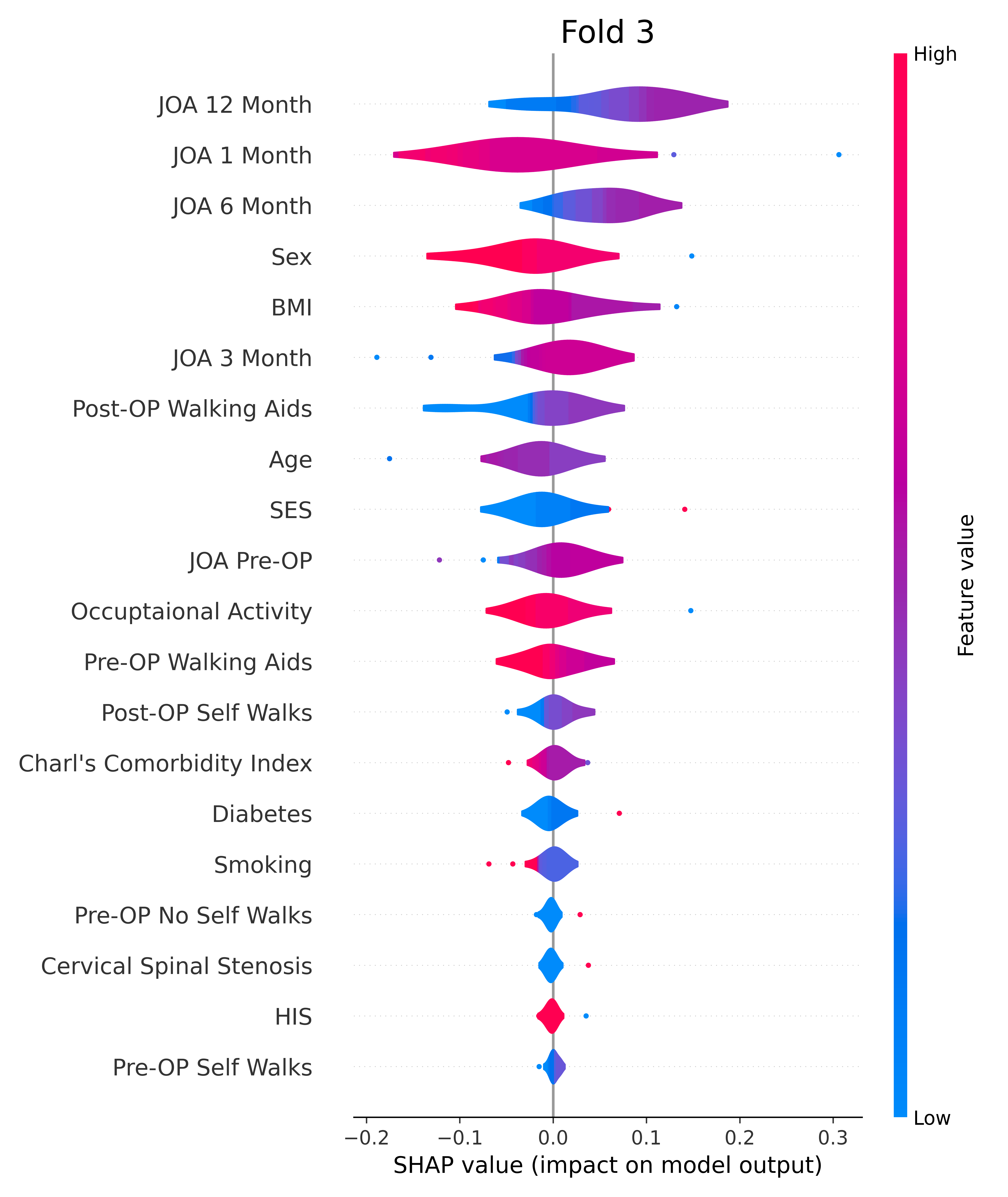


**Figure S10.** **Shapley Additive Explanation (SHAP) values for the LSTM-based model in Fold 3.** The most important feature is the JOA score at 12 months, followed by the JOA score at 1 month, and the JOA score at 6 months. Sex and BMI are also significant contributors, along with the JOA score at 3 months. Post-operative walking aids, SES, the preoperative JOA score, and age complete the top 10 features, underscoring the relevance of both clinical outcomes and patient demographics in predicting long-term postoperative results.


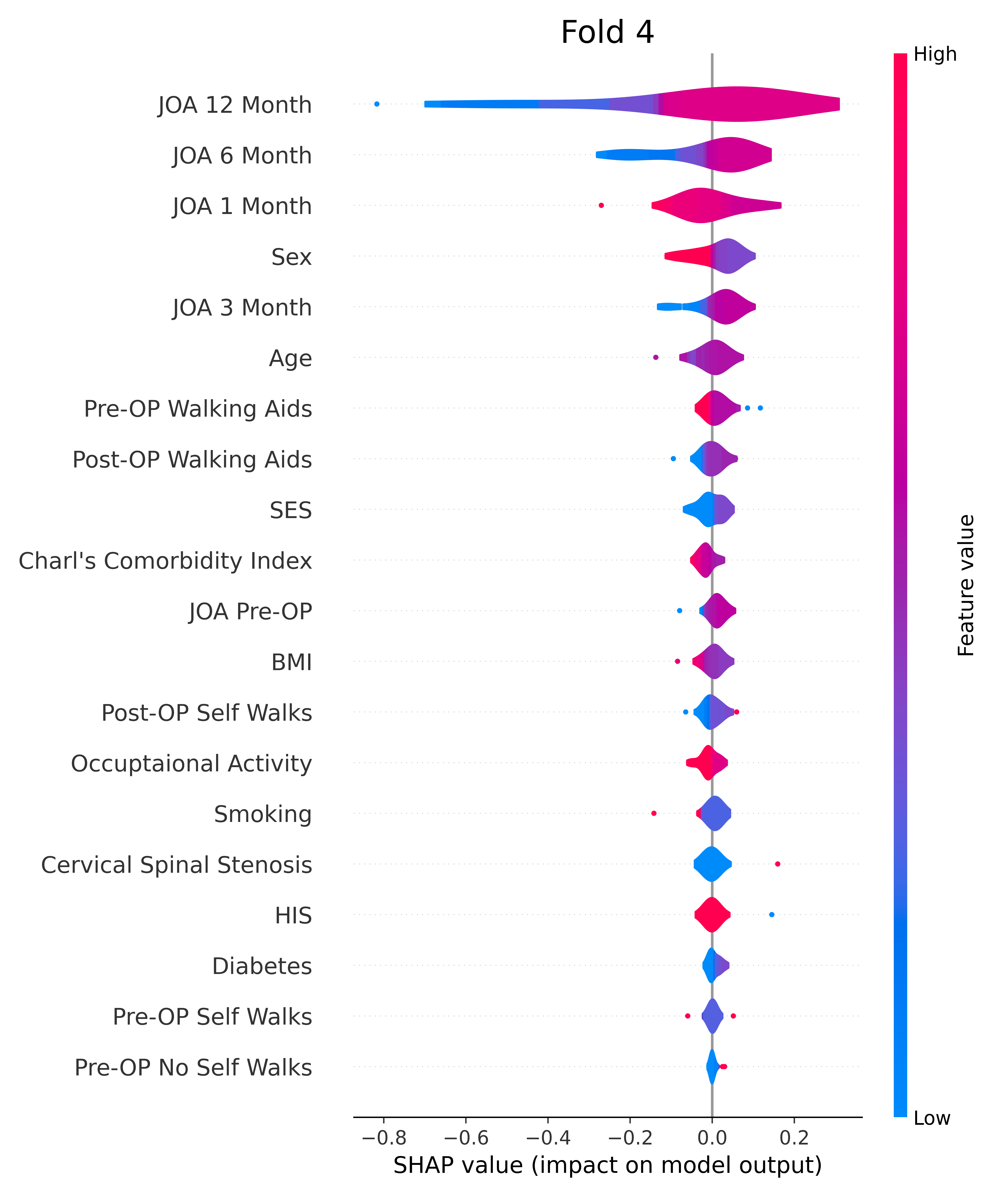


**Figure S11.** **Shapley Additive Explanation (SHAP) values for the LSTM-based model in Fold 4.** The most important feature is the JOA score at 12 months, followed by the JOA score at 6 months, the JOA score at 1 month, and the JOA score at 3 months. Sex and age are also significant contributors, along with pre-operative and post-operative walking aids. SES and the preoperative JOA score round out the top 10 features, underscoring the significance of both clinical progress and patient-specific characteristics in predicting postoperative outcomes.


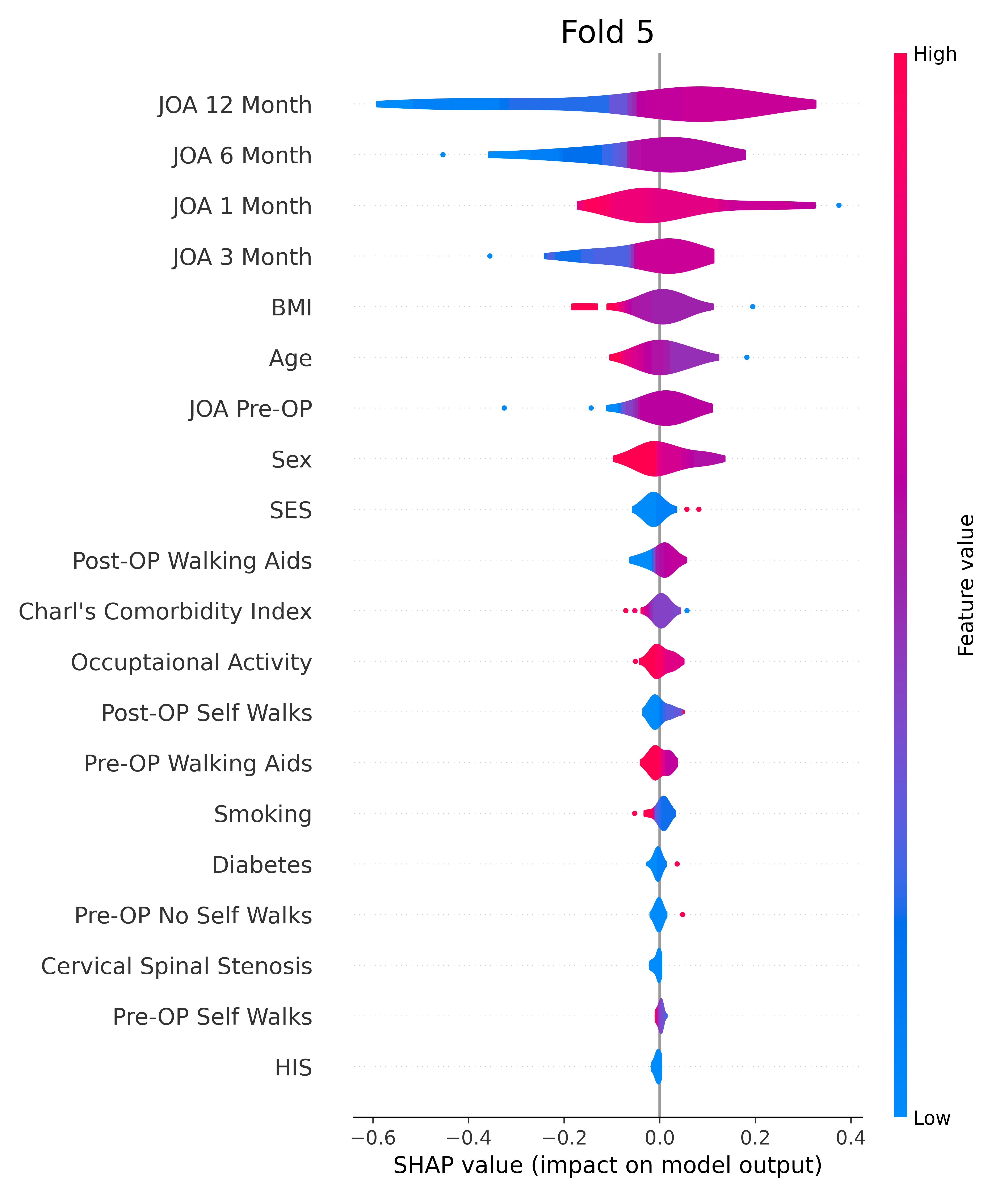
**Figure S12.** **Shapley Additive Explanation (SHAP) values for the LSTM-based model in Fold 5.** The most important feature is the JOA score at 12 months, followed by the JOA score at 6 months, the JOA score at 1 month, and the JOA score at 3 months. BMI, age, and the preoperative JOA score also significantly contribute to the predictions, with sex, SES, and post-operative walking aids completing the top 10 features. This demonstrates the importance of combining clinical outcomes and demographic factors to improve prediction accuracy.
